# Enhancing the efficiency of L-tyrosine by repeated batch fermentation

**DOI:** 10.1080/21655979.2020.1804177

**Published:** 2020-08-11

**Authors:** Guohua Li, Zhichao Chen, Ning Chen, Qingyang Xu

**Affiliations:** a College of Biological Engineering, Tianjin University of Science and Technology, Tianjin, China; b National and Local United Engineering Lab of Metabolic Control Fermentation Technology, Tianjin University of Science and Technology, Tianjin, China

**Keywords:** L-tyrosine, repeated batch fermentation, fed-batch fermentation, productivity, yield

## Abstract

L-tyrosine is a widely used aromatic amino acid with an increasing market demand. Improving the parameters of L-tyrosine production results in a more cost-effective process that is of great interest for industrial applications. *E. coli* GHLTYR-168 was used to ferment L-tyrosine, with a productivity of 1.73 g/(L·h) and a yield of 17.6%. To further increase its production efficiency, repeated batch fermentation was applied to L-tyrosine, in which both replacement time points and ratios were studied during different fermentations. The broth substitution time point had no significant effect on L-tyrosine subjected to repeated batch fermentation, and 70% broth replacement ratio was the best choice. Repeated batch fermentation was performed in 5 batches within 100 h, among which the efficiency of the third batch fermentation was the highest. In the third batch fermentation, the productivity and yield were 2.53 g/(L·h) and 30.1%, respectively. Compared with that during fed-batch fermentation, the productivity and yield of L-tyrosine increased by 43.8% and 74.0%, respectively, during repeated batch fermentation. This is the highest level of L-tyrosine fermentation reported so far. Thus, repeated batch fermentation of L-tyrosine can improve its production efficiency.

## Graphical abstract

**Figure:**
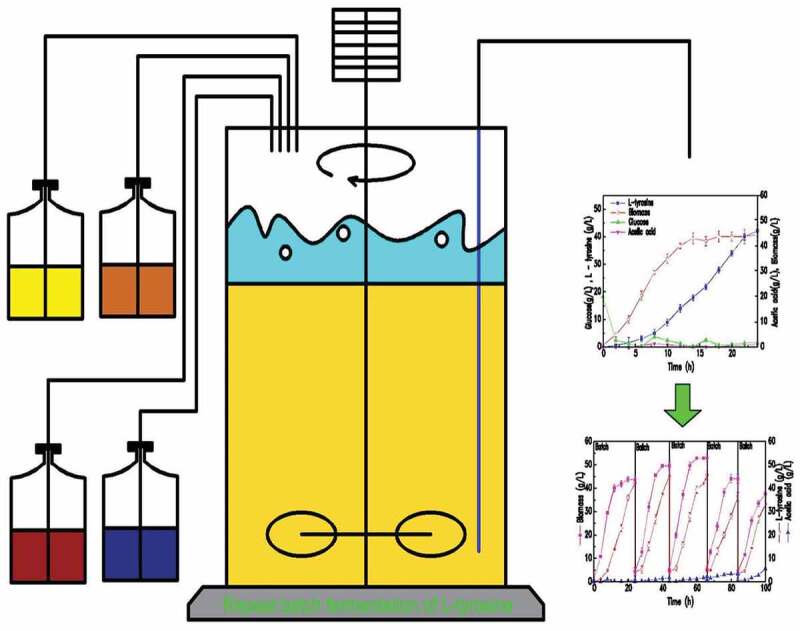

## 1. Introduction

(A) tyrosine has attracted increasing attention due to its special functions in humans and animals. In the food industry, L-tyrosine is used as a nutritional supplement to treat patients with depression [1] and albinism [2]. L-tyrosine is added to animal feed, which can promote the accumulation of fur pigment [3]. It is also a major precursor of neurotransmitters, such as dopamine and thyroxine, which regulate nerve signaling and nervous system development [4]. As an aromatic platform compound, L-tyrosine can be utilized to synthesize high-value drugs, such as resveratrol [5] and tyrosol [6].

The traditional methods of L-tyrosine production are extraction and enzymatic production. Using the extraction method, L-tyrosine was mainly obtained from animal hair, hooves, and horns through acid hydrolysis, neutralization and refining. However, the extraction method is not suitable for large-scale industrial production due to its high cost, low extraction yield, and heavy environmental pollution [7]. Enzymatic methods usually involve the use of tyrosine phenol lyase (TPL) to convert phenol, pyruvate, and ammonia or phenol and L-serine into L-tyrosine [8]. Although the enzymatic production of L-tyrosine has the advantages of strong selectivity and a high conversion rate, poor enzyme stability and activity may restrict its industrial application. In recent years, with increasing attention being paid to environmental protection and sustainable development, L-tyrosine production via microbial fermentation has attracted the attention of researchers. With the development of synthetic biology, L-tyrosine production through microbial fermentation is a viable alternative [9]. After many attempts, 55.54 g/L in a 5-L bioreactor after 40 h of fed-batch fermentation was the highest reported titer of L-tyrosine achieved using microbial fermentation [7]. However, the production cost of this method is too high to satisfy the industrialization of L-tyrosine production by microbial fermentation. In the future, it will be necessary to improve the fermentation efficiency and market competitiveness of this strategy, similar to the other amino acids produced through fermentation, such as glutamate, lysine, and tryptophan.

The fermentation efficiency of L-tyrosine mainly depends on the production strain and fermentation strategy. To the best of our knowledge, most of the L-tyrosine production by microbial fermentation is based on fed-batch fermentation. However, compared with the repeated batch mode, this method is time-consuming and has a low fermentation capacity and high cost [10]. A potential solution to these problems is repeated batch fermentation, which is a high-density fermentation technology that can shorten the non-production time (mainly including seed preparation time) and improve production efficiency [11]. The repeated batch fermentation strategy involves the inoculation of a part or all of the free or immobilized cells into the next batch of fresh medium [12]. In addition, compared with fed-batch fermentation, repeated batch fermentation lasts longer and requires less labor [13]. Economically, repeated batch fermentation increases product yield because only one inoculation is required [10,11]. At the same time, the removal of metabolites from fermented broth reduces the inhibition of the process by metabolites [14]. Despite the significant advantages of this method, repeated batch fermentation has not been reported for tyrosine fermentation. This may be due to the relatively small market demand for L-tyrosine.

Herein, we report for the first time, the production of L-tyrosine using repeated batch fermentation. Based on the characteristics of L-tyrosine production by fed-batch fermentation, the time point and ratio of broth replacement were optimized across three batches of repeated batch fermentation. After optimization, L-tyrosine production using repeated batch fermentation was performed for 5 batches in 100 h under the optimized conditions. Finally, the parameters of L-tyrosine in fed-batch fermentation and repeated batch fermentation were compared.

## 2. Materials and methods

### 2.1. Strains and media

*E. coli* GHLTYR-168 was provided by the Tianjin University of Science and Metabolic Engineering Laboratory. This strain is characterized by W3110, Δ*tnaA*, Δ*mtr, yjiv*::P*trc-aroG, yghx*::P*_trc_-trpE, yyrk*::P*_lac_-trpD*, and *ycjv*::P*_ser_-serA*. The strain was originally designed to produce L-tryptophan, but produced almost no tryptophan. This strain has been patented in China (application no. CN111004761A).

Seed medium: glucose 30 g/L, yeast extract 5 g/L, citric acid 2 g/L, (NH_4_) _2_SO_4_ · 7H_2_O 2.8 g/L, MgSO_4_ · 7H_2_O 9 g/L, V_B1_ 3 mg/L, V_H_ 0.5 mg/L, and trace element solution (TES) 1 mL/L. Fermentation medium: glucose 10 g/L, yeast extract 5 g/L, citric acid 2 g/L, (NH_4_)_2_ SO_4_ 4 g/L, K_2_HPO_4_·H_2_O 15 mg/L, MnSO_4_ · 7H_2_O 10 mg/L, FeSO_4_ · 7H_2_O 2.5 mg/L, MgSO_4_ · 7 H_2_O 1.5 g/L, V_B1_ 1 mg/L, V_H_ 0.5 mg/L, and TES 2 mL/L. TES was comprised of the following (g/L): Al_2_(SO_4_)_3_ · 18H2O, 2.0; CoSO_4_ · 7H_2_O, 0.75; CuSO_4_ · 5H_2_O, 2.5;H_3_BO_3_, 0.5; MnSO_4_·H_2_O, 24; Na_2_MoO_4_ · 2H_2_O, 3.0; NiSO_4_ · 6H_2_O, 2.5; and ZnSO_4_ · 7H_2_O, 15 [15].

### 2.2. Operation method of seed culture and fed-batch fermentation

*E. coli* GHLTYR-168 strains were cultured on agar slants at 36°C overnight. The agar slant medium was the same as the seed medium, but with an additional 25 g/L of agar. The seed culture was prepared to transfer an appropriate amount of agar slant culture to a 5-L fermenter (Baoxing, Shanghai, China) containing 2 L of seed medium. The maximum volume of fermenter was 5 L with a stirring speed of 200–900 rpm. The pH was maintained at 6.9–7.1 by automatically adding NH_4_OH (25%, v/v). The dissolved oxygen was maintained at over 20% by adjusting the stirring speed and aeration rate. The temperature was automatically controlled at 37 ± 0.2°C. When the biomass of the seed culture was about 7 g/L, it was inoculated as a proportion of 15% (v/v) in a 5-L bioreactor (Baoxing, Shanghai, China) containing 3 L of fermentation medium. When the glucose concentration was lower than 2.0 g/L, glucose solution (80%, m/v) was added to ensure a glucose concentration of 0.5–2.0 g/L. The flow rate of glucose solution feeding was calculated using the biomass, as follows: F = (2.9332 × OD + 172.5) g/h. All other methods were performed as previously described.

### 2.3. Operation method of L-tyrosine repeated batch fermentation

Repeated batch fermentation was performed in multiple batches. At the later stage of batch feeding fermentation, a certain proportion of the broth was removed from the fermenter and the same volume of fresh medium was added to the fermenter. In order to determine the most suitable replacement time point for the fermentation broth, three time points were selected from batch fermentation: (i) the time point of maximum fermentation rate (18 h); (ii) the time point of the highest L-tyrosine titer (24 h); (iii) the midpoint of the first two (21 h). In order to determine the optimal ratio of fermented broth replacement (50%, 70%, 80%, or 90% (v/v)), a portion of the old broth was discharged using a peristaltic pump and the same volume of fresh broth was added. The production strains contained in the old fermented broth were inoculated into the next batch of medium as seed cultures. Once the L-tyrosine titer in the previous batch of the fermented broth reached its maximum or increased by less than 5% for two consecutive hours, the next batch of fermentation was started. All of the other fermentation control methods were performed as in fed-batch fermentation.

### 2.4. Analysis method

The biomass was monitored by measuring the absorbance of the culture at 600 nm (OD_600_). In this study, dry cell weight (DCW) (g/L) = 0.364 × OD_600_ [7]. The concentration of residual glucose was measured using an SBA-40E biosensor (Biology Institute of Shandong Academy of Sciences, Jinan, China). The amount of acetic acid and L-tyrosine were measured by high-performance liquid chromatography (HPLC), as described previously [7]. The amount of amino acids in the culture was quantified using an automatic amino acid analyzer (S-433D; Sykam, Germany) according to the manufacturer’s standard operating procedure.

### 2.5. Statistical analysis

All experiments were performed at least in triplicate, and statistical significance was determined using one-way analysis of variance followed by Dunnett’s multiple comparisons test. P < 0.05 was considered statistically significant [16].

## 3. Results and discussion

### 3.1. Fed-batch fermentation of L-tyrosine

**Figure 1.**
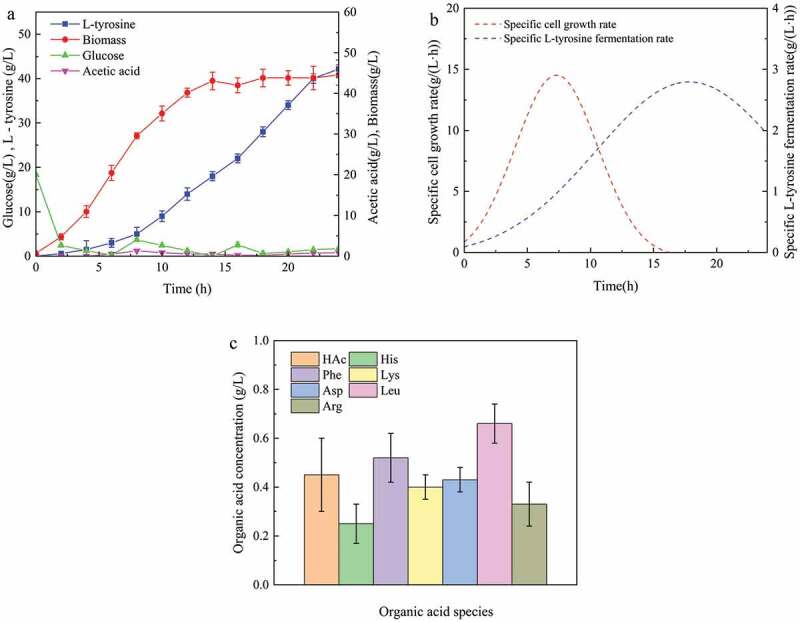
L-tyrosine was produced by *E.coli* GHLTYR-168 batch feed fermentation. (a) Parameters of fed-batch fermentation process; (b) Specific cell growth rate curve and fermentation rate curve; (c) Organic acids in the supernatant of fermentation broth at the end of fed-batch fermentation (24 h).

Fed-batch fermentation is the main method of microbial fermentation currently used in the industry. Figure 1 shows the process curve of L-tyrosine fed-batch fermentation in a 5-L bioreactor. At the earliest, the cells grew rapidly and reached a maximum growth rate of 2.54 g/(L·h) at 8 h, with little L-tyrosine production. Then, L-tyrosine began to be rapidly synthesized in large quantities. The fermentation ability of L-tyrosine gradually increased and reached a maximum fermentation of 2.74 g/(L·h) at 18 h, before gradually decreasing until the end of fermentation. After 24 h of fermentation, the maximum L-tyrosine titer in the fermentation broth was 42.2 g/L. The strain lost its ability to ferment after 24 h, due to cell death, metabolite inhibition, or nutrient deficiency. If cells die, the biomass is likely to decline. However, pre-14 h cells were in the growth phase. After entering the stable period, the biomass increased up to 43 ± 2 g/L (Figure 1(a)). Moreover, once there was no significant change in the concentration of living cells, the fermentation broth was diluted and coated onto LB plates to calculate the number of single colonies [17]. Biomass does not decrease until the end of fermentation, so the decline in fermentation capacity is not caused by cell death. Another possible cause is metabolic feedback inhibition, consisting mainly of target product inhibition (L-tyrosine) and metabolic by-product inhibition (acetic acid) [18,19]. However, because the dissolved L-tyrosine (low solubility easy to crystallize) and acetic acid (less than 0.5 g/L (Figure 1(a,c)) concentrations were very low, metabolite feedback inhibition was discarded. Therefore, the most likely cause of the loss of 24 h fermentation capacity is nutrient deficiency [20]. In addition, there are only 6 amino acids in the fermentation broth (at the end of fermentation) at concentrations above 0.1 g/L, and all are below 1 g/L (Figure 1(c)). This also indicates a lack of nutrients in the later stages of fermentation. In addition, high biomass and high metabolic activity bring dissolved oxygen close to 0 (data not shown) during stable periods. Moreover, L-tyrosine is very easy to crystallize because of its very low solubility. When the amount of L-tyrosine crystals is large, there will be a large amount of foam, causing a decrease in oxygen transfer [21]. This is an important reason for the reduction in fermentability.

The fermentation rate and the cell growth rate are not synchronous (Figure 1(b)), and the L-tyrosine fermentation process belongs to the non-growth coupling type. The fermentation rate is faster when the biomass is large. Since the residual glucose concentration in the fermentation broth is always lower than 2 g/L, the acetic acid concentration level is relatively low (Figure 1(a)). The glucose feeding strategy was suitable and the synthesis of metabolic by-products was reduced. In conclusion, the fed-batch fermentation productivity of L-tyrosine was 1.76 g/(L ·h) with a yield of 17.3%.

In a recent study, *E. coli* HRP was fermented by batch feeding in a 3-L fermentation tank for 40 h, giving rise to a L-tyrosine titer of 55.54 g/L and a productivity of 1.38 g/(L·h) [7]. Although this is the highest level of L-tyrosine titer reported, the fermentation reported in this paper was higher. The main reason may be the biomass. Here, the biomass of *E. coli* GHLTYR-168 was 43.0 g/L, while the maximum biomass of *E. coli* HRP was only 24 g/L. Moreover, the cell growth period of *E. coli* GHLTYR-168 was short (only 14 h). *E. coli* HRP, however, had a long cell growth period of at least 20 h. It is generally believed that for non-growth coupled fermentation, high biomass helps to improve fermentation ability. On the other hand, high biomass means that more nutrients are used for cell growth rather than for synthetic products. The yield of *E. coli* HRP was 25.2%, that is 7.9% higher than that of *E. coli* GHLTYR-168. A higher titer provides greater economic benefits in the industrial application of microbial fermentation. Therefore, the fermentation efficiency of *E. coli* GHLTYR-168 will need to be improved further. There are several potential strategies to increase productivity. For example, L-tyrosine inhibition can be relieved if L-tyrosine crystals in fermented broth are isolated in time. If the cell growth period is shortened, the fermentation ability of L-tyrosine can also be enhanced. Moreover, it is possible to increase productivity by supplementing the medium with fresh medium when fermentability declines. In this context, repeated batch fermentation is a potential option.

### 3.2. L-tyrosine is produced by repeated batch fermentation

Repeated batch fermentation experiments involve using fresh sterile broth instead of the old broth (or culture medium) at the appropriate time during the batch fermentation process. According to previous reports, repeated batch fermentation can improve the fermentability and titer of 1,3-PDO compared with batch fermentation [22]. *S. cerevisiae* (ATCC 36,858) repeated batch fermentation for ethanol production has been shown to reduce the fermentation time and improve the yield [23].

#### 3.2.1. Effect of replacement time points on L-tyrosine repeated batch fermentation

To determine the effect of fermentation broth replacement time points on the repeated batch fermentation of L-tyrosine, three special time points were selected for repeated batch fermentation in a 5-L fermentation tank for 18 h (maximum fermentation rate), 21 h (midpoint of 18 h and 24 h), and 24 h (L-tyrosine titer and maximum fermentation over). Eighty percent of the fermentation broth was replaced by fresh medium, and three batches of fermentation were performed.

**Figure 2.**
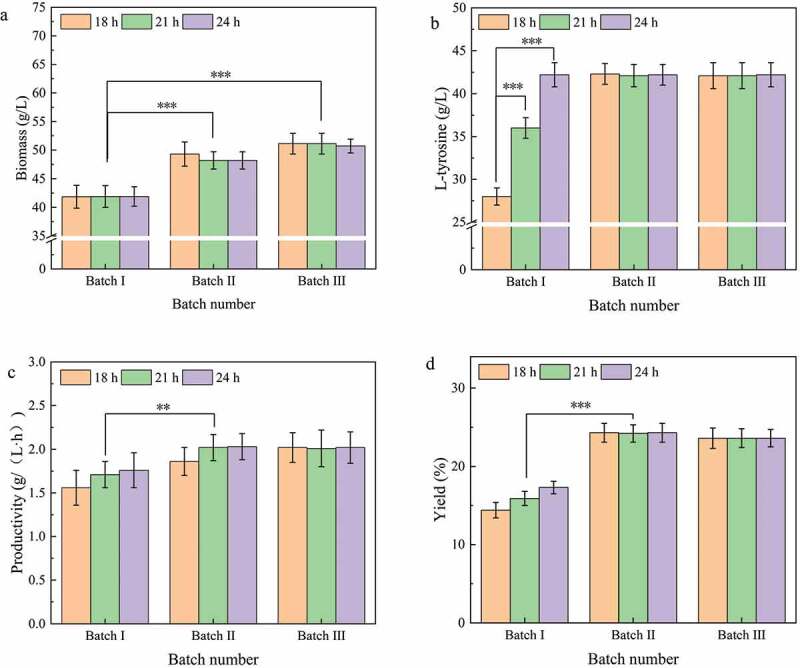
Effect of time points of replacement of fermentation broth of L-tyrosine production by repeated batch fermentation.

According to the experimental results (Figure 2), repeated batch fermentation had a positive effect on L-tyrosine, but different fermentation broth replacement time points were not significantly different between the fermentation batches. Compared to the first batch of batch fermentation, the maximum biomass in the second and third batches was 49.3 g/L and 51.1 g/L, respectively (Figure 2(a)), which were 17.4% and 21.7% higher than fed-batch fermentation. The second and third batches were the same as fed-batch fermentation in the final L-tyrosine titer of 42.2 g/L (Figure 2(b)). The second and third batches had a higher productivity and yield than the first batch (Figure 2(c,d)). Therefore, the efficiency of repeated batch fermentation is higher than fed-batch fermentation. These results are in line with those obtained from the repeated batch fermentation of other products [24]. However, there was no significant difference in the effect of different fermentation broth replacement time points on repeated batch fermentation. Although similar studies have been reported in repeated batch fermentation of exopolysaccharide, the conclusions have varied [25]. In exopolysaccharide fermentation, the efficiency of cell repeated batch fermentation was low due to an accumulation of harmful metabolic by-products. However, due to the good glucose feeding strategy adopted in this experiment, the concentration of acetic acid, the main metabolic by-product, was very low (Figure 1(c)) and did not inhibit cell activity. The decline of cell fermentation ability during batch fermentation was mainly due to nutrient deficiency. Cell growth and fermentation capacity were restored by supplementing fresh medium during repeated batch fermentation. In summary, in consideration of the fermentation efficiency of the first batch (fed-batch fermentation), the next batch fermentation was performed by replacing the fermentation broth with a fresh medium at 24 h. Moreover, at the end of the last batch of fermentation (L-tyrosine titer does not increase), the fresh culture medium was replaced by the old culture medium to begin the next batch of fermentation.

#### 3.2.2. Effect of broth replacement ratio on L-tyrosine in repeated batch fermentation

The broth replacement ratio plays an important role in determining the operation of repeated batch fermentation by adjusting the amount of biomass and fresh culture medium added to the next batch. In this section, different replacement ratios of fermentation broth were studied. When the L-tyrosine titer was no longer increased in the fermented broth, three batches of repeated fermentation were performed using fresh medium to replace the fermented broth at a ratio of 50%, 70%, 80%, and 90%.

**Figure 3.**
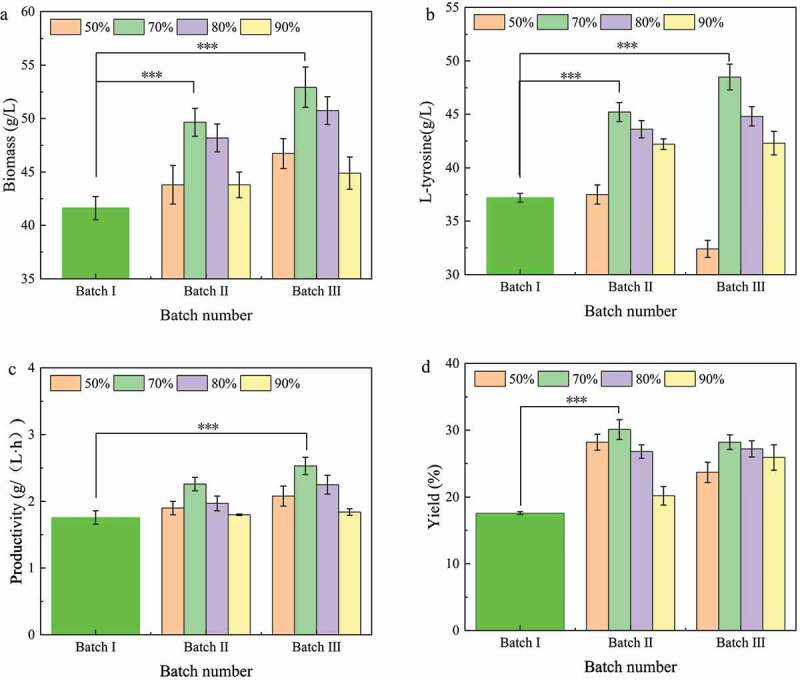
Effect of fermentation broth replacement ratio on L-tyrosine production by repeated batch fermentation.

As shown in Figure 3(a), the biomass of the second and third batches in repeated batch fermentation increased with the ratio of fermentation broth replacement, of which 70% was the highest. The L-tyrosine titers were increased in the second and third batches at 70% and 80% broth replacement ratios, with 70% being the highest (Figure 3(b)). The L-tyrosine titer of 90% broth replacement ratio remained unchanged in the second and third batches, while the L-tyrosine titer of 50% decreased (Figure 3(b)). The final 70% of fermentation broth replacement ratio produced the highest productivity and yield in the second and third batches of fermentation (Figure 3(c,d)). In conclusion, the replacement ratio of 70% was found to be the best choice for repeated batch fermentation of L-tyrosine.

#### 3.2.3. Effect of repeated batch fermentation on L-tyrosine

**Figure 4.**
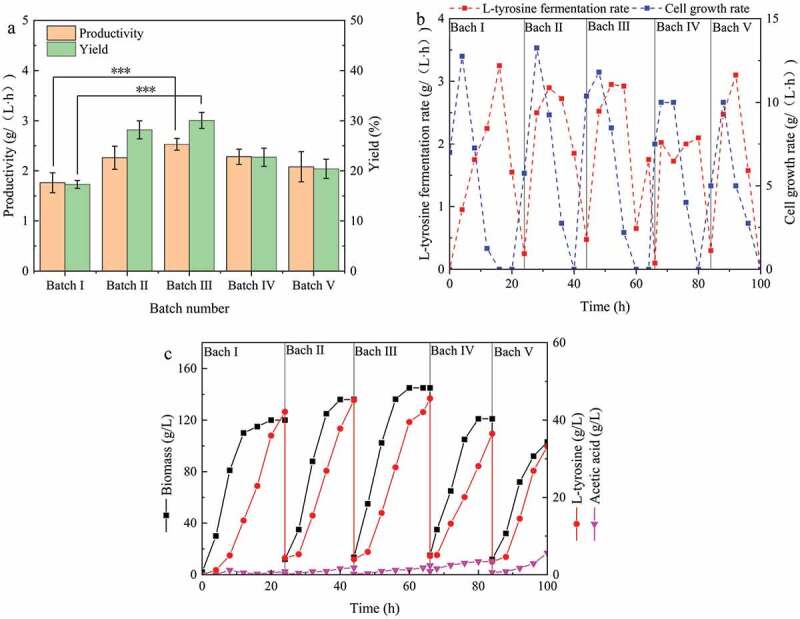
Repeated batch fermentation for L-tyrosine production. (a) Different batches of productivity and yield; (b) Fermentation rate and cell growth rate curves of different batches; (c) Parameters of repeated batch fermentation process.

According to these optimized conditions, when the L-tyrosine titer no longer increased in each batch of fermentation and 70% of the fermentation broth was replaced, repeated batch fermentation was performed within 100 h. The experimental results are shown in Figure 4.

The fermentation period from the first batch to the fifth batch was 24 h, 20 h, 18 h, 16 h, and 16 h, respectively (Figure 4(c)). In repeated batch fermentation, the fermentation period was gradually shortened, with the maximum fermentation period shortened by 33.3%. In the literature, the shortening of fermentation period has been reported to result in high-density cell cultures [23]. In the present study, the biomass increased from the first batch to the third batch, and then decreased (Figure 4(c)). The biomass of the third batch increased to 52.9 g/L, and that of fed-batch fermentation (the first batch) increased by 20.8%. These results indicate that repeated batch fermentation is a high-density cell fermentation method, and provides the same results as other products [26]. In addition, the biomass increased rapidly in new batch fermentation after supplementing with fresh medium (Figure 4(b,c)). This indicates that the inhibition of the substrate on cell growth can be ignored since the rapid growth period follows a short adaptation period.

The trend of productivity was the same as that of biomass. In the third batch, the highest productivity was 2.53 g/(L·h) (Figure 4(a)), which was 43.8% higher than that obtained using fed-batch fermentation (in the first batch). Similar findings have been reported by Moeller et al., who found that the biomass and citric acid titer was highest in the third batch of fermentation [27]. During the first three batches of fermentation, the L-tyrosine titer and yield gradually increased, reaching a maximum value of 45.6 g/L and 30.1% (Figure 4(a)), respectively. In addition, the L-tyrosine titer curve showed that there was an initial delay in the synthesis of L-tyrosine in each batch (Figure 4(b,c)). For example, in the second batch of fermentation, the cell growth rate and fermentation rate were 5.75 g/(L·h) and 0.25 g/(L·h) at 24 h, 43% of the maximum growth rate (28 h), and 8% of the maximum fermentation rate (32 h), respectively (Figure 4(b)). The difference between 43% and 8% showed different levels of cell growth and fermentation inhibition by substrates, possibly due to substrates inhibiting L-tyrosine synthesis [28]. A high concentration of substrates can cause cellular stress and have harmful effects on metabolism [29]. This is inconsistent with previous reports that high concentrations of substrates inhibit cell growth and productivity [30]. However, the high concentration of substrate in this experiment only inhibited productivity and not cell growth (Figure 4(b)). This inhibition can be resolved by pulse feeding or continuous culturing. After the fourth batch, the productivity and yield were found to decline, mainly due to a decrease in the cell viability due to a prolonged culture. In aerobic cultures, cells are particularly easy to senescence, and metabolic activity is prone to decline. In addition, long-term cultivation increased the risk of contamination [31].

The concentration of acetic acid in the first two batches was lower than 2.0 g/L (Figure 4(c)), while the acetic acid concentrations in batches 3, 4, and 5 were 2.4 g/L, 3.4 g/L, and 5.6 g/L, respectively. Moreover, the concentration of acetic acid in the fermentation broth was found to increase gradually. Yang et al. [22] found that the levels of metabolic by-products (lactic acid and ethanol) increased in batches and inhibited cell growth and fermentation during the process of repeated batch fermentation for the production of 1,3-propanediol. These findings are consistent with those obtained in the present study. It is generally believed that the concentration of acetic acid above 2 g/L will inhibit cell growth and metabolism. Thus, after the third batch fermentation, decreases in biomass, productivity, and yield were associated with an excessive accumulation of acetic acid. Under the above glucose feeding strategy, the glucose consumption rate of unit bacteria was found to be more or less consistent throughout the fermentation process.

It is worth mentioning that this is the first report to produce L-tyrosine by repeated batch fermentation. The productivity and yield of L-tyrosine were 2.53 g/(L·h) and 30.1% during the repeated batch fermentation of *E. coli* GHLTYR-168, which were 43.8% and 74.0% higher than that during fed-batch fermentation. This is the highest reported level of productivity and yield (Table 1).

**Table 1.** Fermentation of L-tyrosine by different microorganisms and culture methods.

Microorganisms	Titer(g/L)	Yields(%)	Productivity (g/(L·h))	Cultivation mode	Reference
*E.coil* K12	55	9	1.15	Fed-batch	[9]
*C. Glutamicum* KY10865	26	–	0.33	Fed-batch	[35]
*E.coil* HRP	55.54	25	1.38	Fed-batch	[7]
*E.coli* GHLTYR-168	42.2	17.3	1.76	Fed-batch	This study
*E.coli* GHLTYR-168	45.6	30.1	2.53	Repeated-batch	This study

According to a large number of studies, fermentation shows a higher fermentation efficiency under continuous mode [32]. Continuous modes include chemostat, cell cycle, and cell immobilization, among others. Aso et al. used cell cycle fermentation to improve the productivity of lactic acid by 4-fold that of batch fermentation [33]. Cell recycling increased the productivity of butanol production by 6-fold that of high cell density fermentation [34]. Further research on these aspects will be conducted in future studies to improve the fermentation efficiency of L-tyrosine.

## 4. Conclusion

In this study, L-tyrosine was produced for the first time by repeated batch fermentation, and the highest levels of production were reported. When L-tyrosine titers in the previous batch of fermented broth reached their maximum, 30% of the broth was inoculated into the next batch of fermentation for repeated batch fermentation. The productivity and yield of L-tyrosine by repeated batch fermentation of *E. coli* GHLTYR-168 increased by 43.8% and 74.0%, respectively, compared with fed-batch fermentation.

## Highlights

- Repeated batch fermentation was used in L-tyrosine production.
- The yield of L-tyrosine increased by as much as 74.0%.
- Productivity rose by a maximum of 43.8%.
